# Apply pressure-strain loop to quantify myocardial work in pulmonary hypertension: A prospective cohort study

**DOI:** 10.3389/fcvm.2022.1022987

**Published:** 2022-12-15

**Authors:** Jian Wang, Chao Ni, Menghui Yang, Xueming Zhang, Binqian Ruan, Lingyue Sun, Xuedong Shen, Jieyan Shen

**Affiliations:** ^1^Department of Cardiology, Renji Hospital, Shanghai Jiao Tong University School of Medicine, Shanghai, China; ^2^Department of Cardiology, Ningbo First Hospital, Ningbo Hospital of Zhejiang University, Ningbo, China; ^3^Children’s Heart Center, Institute of Cardiovascular Development and Translational Medicine, The Second Affiliated Hospital and Yuying Children’s Hospital of Wenzhou Medical University, Wenzhou, China

**Keywords:** pressure-strain loop, pulmonary hypertension, myocardial work, cardiac function, prognosis

## Abstract

**Objectives:**

Pressure-strain loop (PSL) is a novel method to quantify myocardial work in many cardiovascular diseases. To investigate the value of myocardial work parameters derived from PSL for evaluating cardiac function and clinical prognosis in patients with pulmonary hypertension (PH).

**Methods:**

A total of 52 patients with PH and 27 healthy controls were enrolled in this prospective study. PSLs determined by echocardiography were used to calculate global work index (GWI) of left ventricle (LV) and right ventricle (RV). Global constructive work (GCW) comprised the sum of myocardial work performed during shortening in systole and during lengthening in isovolumic relaxation. Global wasted work (GWW) comprised the sum of myocardial work performed during lengthening in systole and during shortening in isovolumic relaxation. Global work efficiency (GWE) was defined as GCW/(GCW + GWW).

**Results:**

LVGWW, RVGWI, RVGCW and RVGWW were significantly higher in patients than controls (all *P* < 0.001). LVGWE, LVGWI, LVGCW, and RVGWE were lower in patients than controls (all *P* < 0.01). Myocardial work parameters correlated well with clinical and other conventional echocardiographic assessments (all *P* < 0.05). In binary logistic regression analysis, the combination of RVGWE and estimation of pulmonary arterial systolic pressure (ePASP) was the best model to predict clinical outcomes (OR = 0.803, *P* = 0.002 and OR = 1.052, *P* = 0.015, respectively). Receiver operating characteristic curv demonstrated the combination of RVGWE and ePASP was the best predictor of adverse events with 100% sensitivity and 76.3% specificity (AUC = 0.910, *P* < 0.001).

**Conclusion:**

Myocardial work parameters derived from PSL are emerging markers of cardiac function. And the combination of RVGWE and ePASP is a useful predictor of clinical outcome in PH patients.

## Introduction

Pulmonary hypertension (PH) is a pathological state or disease caused by abnormal increase of pulmonary circulation pressure due to various factors or diseases (1). According to the results from a National Prospective Registry, the median survival of PH patients is 2.8 years (95% CI 1.9–3.7 years) in the absence of effective treatment (2).

It is well acknowledged that cardiac function is directly related to the prognosis of patients. A great number of studies have shown that PH patients in World Health Organization functional class (WHO-FC) IIIWH have worse outcomes in the long-term prognosis (3, 4). Previous studies on PH have mostly focused on the right ventricular (RV) function, as long-term RV afterload will directly cause RV dysfunction which is the major determinant of survival in these patients (5, 6). However, recently, scholars find that left ventricle (LV) is also impaired in PH patients despite of normal LV ejection fraction (LVEF) and LV dysfunction is associated well with poor clinical outcomes (7, 8). Therefore, both RV and LV function should be concentrated on equally in PH patients.

Echocardiography is widely used to evaluate cardiac function in PH patients at the advantage of non-invasion (1). Global longitudinal strain (GLS) has proven benefit for assessing both LV function and RV function. Yet GLS is load dependency and is not adjusted for afterload, which may influence the accuracy of cardiac function evaluation (9), especially in PH patients at high level of afterload. Recently, Russell et al. (10) propose a novel non-invasive method by quantifying myocardial work to assess ventricular systolic function, which is termed as pressure-strain loop (PSL). It takes account of GLS data with non-invasive estimated ventricular pressure curves simultaneously and the area within PSL represents myocardial work. Study on hypertension has shown that LV myocardial work parameters are significantly higher in moderate to severe hypertension patients while LVGLS are preserved compared to controls (11).

No previous study has assessed myocardial work in PH patients. Thus, in this study, we aimed to quantify LV and RV myocardial work in PH patients by non-invasive PSL, and explore the value of myocardial work parameters of evaluating cardiac function and clinical prognosis.

## Materials and methods

### Study population

Fifty-two patients diagnosed with PH between January 2019 and June 2020 in Shanghai Renji Hospital were consecutively enrolled in this prospective study. Given PH prevalence and incidence were mostly in middle-aged women, we recruited twenty-seven age- and gender- matched healthy controls from medical center for further study. According to 2015 ESC/ERS Guidelines of PH (1), the diagnosis of all patients was mean pulmonary arterial pressure (mPAP) ≥ 25 mmHg, pulmonary artery wedge pressure (PAWP) ≤ 15 mmHg and pulmonary vascular resistance (PVR) >3 Wood unit at rest detected by right heart catheterization (RHC), including type I, type IV and type V pulmonary hypertension. Exclusion criteria were listed as the following: PH due to left heart disease; PH due to hypoxia; arrhythmia (atrial fibrillation or flutter, left or right bundle branch block, et al.); pregnancy; cancer; patients with obscure endocardium in echocardiography; lost to follow-up.

### Clinical data collection

Clinical data of PH patients were acquired by reviewing electronic medical records. Age, gender, etiological classification, brain natriuretic peptide (BNP), RHC data including pulmonary arterial systolic pressure (PASP), pulmonary arterial diastolic pressure (PADP), mPAP, PAWP, cardiac output (CO), cardiac index (CI) and PVR, as well as the specific drug therapy were recorded from all patients. Patients underwent RHC and echocardiography within 3 days of each other. Before echocardiography, all patients’ blood pressure (BP) by a brachial artery cuff were measured immediately. In order to evaluate the cardiac function, all patients underwent 6-minute walking distance (6MWD) to identify function capacity and functional class was determined by clinical investigator based on the WHO-FC (12).

### Echocardiography

Standard transthoracic echocardiography connecting with electrocardiogram were performed in all of the 52 PH patients and 27 controls using a Vivid E95 ultrasound machine (GE Healthcare, Horten, Norway) equipped with an M5S probe by an experienced doctor. As recommended by the American Society of Echocardiography (13), images in parasternal long-axis, apical four-chamber, apical two-chamber and apical long-axis views were acquired. All the images were transferred from the machine at least three consecutive beats, and then offline measured using EchoPAC (version 203, GE Healthcare, Horten, Norway) by another independent echocardiographer who did not take part in the image acquisition and was blinded to clinical data. LVEF was measured by using Simpson’s technique. Tricuspid annular plane systolic excursion (TAPSE) was obtained by an M-mode cursor oriented to the junction of the RV free wall and the tricuspid valve plane. RV fractional area change (FAC) was calculated as following formula: (RV end-diastolic area – RV end-systolic area)/RV end-diastolic area × 100%. Right atrial area (RAA) was measured in end-systole. Right ventricle and left ventricle basal diameter were measured to get right ventricle/left ventricle basal diameter ratio (RV/LV). In the absence of pulmonic valve or right ventricular outflow tract stenosis, the estimation of PASP (ePASP) was equal to right ventricular systolic pressure (RVSP), which was calculated by adding tricuspid regurgitation peak gradient to the right atrial pressure (RAP). RAP was estimated by observing inferior vena cave (IVC) diameter and its collapse during inspiration.

### Ventricular global longitudinal strain and myocardial work analysis

Images from apical four-chamber, apical two-chamber and apical long axis views were put into offline workstation (EchoPAC version 203, GE Healthcare, Horten, Norway) to yield LV global longitudinal strain (LVGLS). RV global longitudinal strain (RVGLS) was assessed by using an apical four-chamber view focusing on the RV in offline workstation.

**FIGURE 1:**
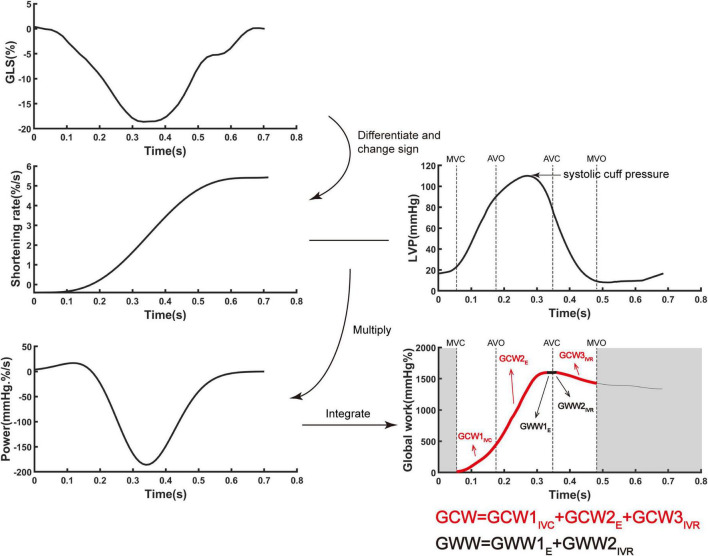
The algorithm of ventricular myocardial work by incorporating global longitudinal strain and ventricular pressure. Myocardial work was quantified by calculating the rate of segmental shortening by differentiating the strain curve and multiplying the resulting value by the instantaneous ventricular pressure. E, ejection period; IVC, isovolumic contraction period; IVR, isovolumic relaxation period; GCW, global constructive work; LVP, left ventricular pressure; GLS, global longitudinal strain; GWW, global wasted work.

Ventricular myocardial work was quantified by a novel non-invasive PSL method which used GLS combined with estimated non-invasive pressure (10). The PW Doppler signal of the LV outflow tract was used to set marker of aortic valve closure time. As referenced to Russel et al. (10), the ventricular pressure was estimated using a standard pressure trace which was personalized by stretching it in time according to valvular event times (mitral valve open/close and aortic valve open/close) by echocardiography and in amplitude according to measured systolic artery pressure. Myocardial work or global work index (GWI) was calculated as the integral of power from mitral valve close to mitral valve open which was generated by differentiating the strain curve over time, giving the myocardial shortening rate, and then multiplying this with instantaneous ventricular pressure (Figure 1). Global constructive work (GCW) comprised the sum of myocardial work performed during shortening in systole and during lengthening in isovolumic relaxation, whereas global wasted work (GWW) comprised the sum of myocardial work performed during lengthening in systole and during shortening in isovolumic relaxation (14). Global work efficiency (GWE) was defined as GCW divided by the sum of GCW and GWW, expressed as a percentage.

LV pressure was replaced by the cuff systolic pressure. Because of the particularity of RV, in order to improve the accuracy of pulmonary arterial systolic pressure obtained by echocardiography, other right cardiac function parameters obtained from echocardiography, including ePASP, TAPSE, FAC, RAP, RAA, RV/LV, LVGLS and RVGLS, were used for adjusting, then the calculated pulmonary arterial systolic pressure (PASPcal) were obtained. In the absence of left/right bundle branch block, the left and right heart could be considered as synchronous contraction. Similarly, the estimation of real time RV pressure was acquired by using an empiric reference curve which was adjusted by stretching it in time according to valvular event times (mitral valve open/close and aortic valve open/close) by echocardiography and in amplitude according to PASPcal. LVGWE, LVGWI, LVGCW, LVGWW, RVGWE, RVGWI, RVGCW, and RVGWW were eventually obtained by the algorithm above-mentioned.

The interobserver reliability of GLS and myocardial work parameters was assessed using the measurements of 10 randomly chosen PH patients and 5 randomly chosen controls by two experienced cardiologists.

### Follow-up

All patients with PH were followed up regularly at 3-month intervals by telephone calls to ascertain present symptom, therapy, and cardiac function. The deadline of the last visit of the last patient was May 2021. Major adverse events defined as all-cause of mortality, hospitalization and need of new specific drug therapy or enhancement on the original therapy basis were recorded.

### Statistical analysis

Normally distributed continuous variables were presented as mean ± standard deviation, and non-normally distributed continuous variables as median (first and third quartiles). Categorical variables were expressed as numbers (percentage). Independent sample *t*-test or Mann-Whitney *U* test was used to compare variables between PH patients with controls, while Pearson’s Chi-Squared test or Fisher exact test to compare the categorical variables (15). PASPcal was adjusted for TAPSE, FAC, RAP, RAA, RV/LV, LVGLS and RVGLS by multiple linear regression, and Bland-Altman plot was used for agreement analysis. And the Spearmen correlation coefficients were tested to analyze the relationship between BNP, 6MWD, WHO-FC, TAPSE, FAC, RAA, RV/LV with myocardial work parameters in PH patients. At the end of follow-up, we performed binary logistic regression and receiver operating characteristic (ROC) curve to predict the risk factor of adverse events. Hosmer-Lemeshow test was used to test the fitting degree of logical regression. Statistical analysis was performed with SPSS 21.0 (IBM Corp., Armonk, NY, USA). Figures were performed with MATLAB (R2020, Mathworks, Nattick, USA) and GraphPad Prism 8 (GraphPad Software, La Jolla, CA). For all tests, a 2-tailed *P* value of < 0.05 was considered to be statistically significant.

## Results

### Main clinical characteristics of pulmonary hypertension patients and controls

A total of 52 PH patients and 27 controls (female 84.6 vs 85.2%, *P* = 0.947; age 44.1 ± 13.2 vs 43.0 ± 13.7 years, *P* = 0.748) were included in this clinical study. Main clinical characteristics of patients were shown in Table 1.

**TABLE 1:** Baseline clinical characteristics of pulmonary hypertension patients.

Characteristic	PH (*n* = 52)
Gender (% female)	44 (84.6)
Age (years)	44.1 ± 13.2
SBP (mmHg)	120.4 ± 18.1
DBP (mmHg)	75.7 ± 13.4
BNP (pg/ml)	188.0 (44.5,435.5)
6MWD (m)	430.6 ± 109.7
**WHO-FC (%)**	
I	15 (28.8)
II	16 (30.8)
III	18 (34.6)
IV	3 (5.8)
**Etiology (%)**	
Connective tissue disease associated with pulmonary arterial hypertension	33 (63.5)
Idiopathic pulmonary arterial hypertension	5 (9.6)
Congenital heart disease associated with pulmonary arterial hypertension	4 (7.7)
Portopulmonary hypertension	1 (1.9)
Chronic thromboembolic pulmonary hypertension	6 (11.5)
Pulmonary hypertension with unclear and/or multifactorial mechanisms	3 (5.8)
**Specific drug therapy**	
None (%)	5 (9.6)
Endothelin receptor antagonists (%)	35 (67.3)
Phosphodiesterase type 5 inhibitors (%)	35 (67.3)
Riociguat (%)	3 (5.8)
Prostacyclin analogues (%)	7 (13.5)
**RHC data**	
Pulmonary arterial systolic pressure (mmHg)	72.9 ± 18.0
Pulmonary arterial diastolic pressure (mmHg)	34.8 ± 8.3
Mean pulmonary arterial pressure (mmHg)	48.8 ± 10.5
Pulmonary artery wedge pressure (mmHg)	10.5 ± 3.8
Cardiac output (L/min)	4.6 ± 1.6
Cardiac index (L/min⋅m^2^)	2.9 ± 0.9
Pulmonary vascular resistance (dyn⋅*s*⋅cm^–5^)	785.8 ± 403.9

Values are mean ± SD, *n* (%), or median (first and third quartiles).

6MWD, 6-minute walking distance; BNP, brain natriuretic peptide; DBP, diastolic blood pressure; PH, pulmonary hypertension; RHC, right heart catheterization; SBP, systolic blood pressure; WHO-FC, World Health Organization functional class.

### Echocardiography and myocardial work parameters

**FIGURE 2:**
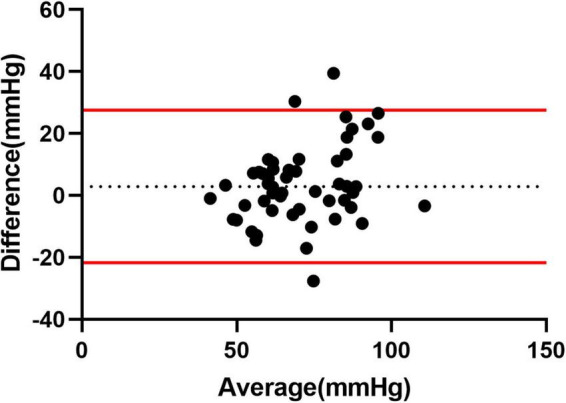
Agreement analysis between PASPcal and PASP from RHC. Bland-Altman plot showed great agreement between PASPcal and PASP (mean bias 3.554 mmHg, 95% limits of agreement −21.17 to 28.27 mmHg). LV-PSL, left ventricular pressure-strain loop; RV-PSL, right ventricular pressure-strain loop.

Multiple linear regression showed that PASPcal had a good correlation with PASP from RHC (*R*^2^ = 0.560, *P* < 0.001). The formula was as follows: PASPcal = 0.609*ePASP + 0.187*TAPSE − 0.182*FAC + 0.153* RAP + 0.12*RAA − 0.128*RV/LV − 0.153*LVGLS + 0.135* RVGLS. Bland-Altman plot also showed great agreement between PASPcal and PASP (Figure 2). Intra-class correlation coefficients of GLS and myocardial work parameters were shown in Table 2.

**TABLE 2:** Intra-class correlation coefficient of global longitudinal strain and myocardial work parameters.

	ICC	95%CI	*P*-value
LVGLS	0.961	0.883–0.987	< 0.001
RVGLS	0.981	0.944–0.994	< 0.001
LVGWE	0.897	0.694–0.966	< 0.001
LVGWI	0.939	0.818–0.979	< 0.001
LVGCW	0.923	0.770–0.974	< 0.001
LVGWW	0.889	0.669–0.963	< 0.001
RVGWE	0.804	0.416–0.934	0.002
RVGWI	0.982	0.945–0.994	< 0.001
RVGCW	0.990	0.969–0.997	< 0.001
RVGWW	0.815	0.449–0.938	0.002

CI, confidence interval; ICC, intra-class correlation coefficient; LVGCW, left ventricular global constructive work; LVGLS, left ventricular global longitudinal strain; LVGWE, left ventricular global work efficiency; LVGWI, left ventricular global work index; LVGWW, left ventricular global wasted work; RVGCW, right ventricular global constructive work; RVGLS, right ventricular global longitudinal strain; RVGWE, right ventricular global work efficiency; RVGWI, right ventricular global work index; RVGWW, right ventricular global wasted work.

**FIGURE 3:**
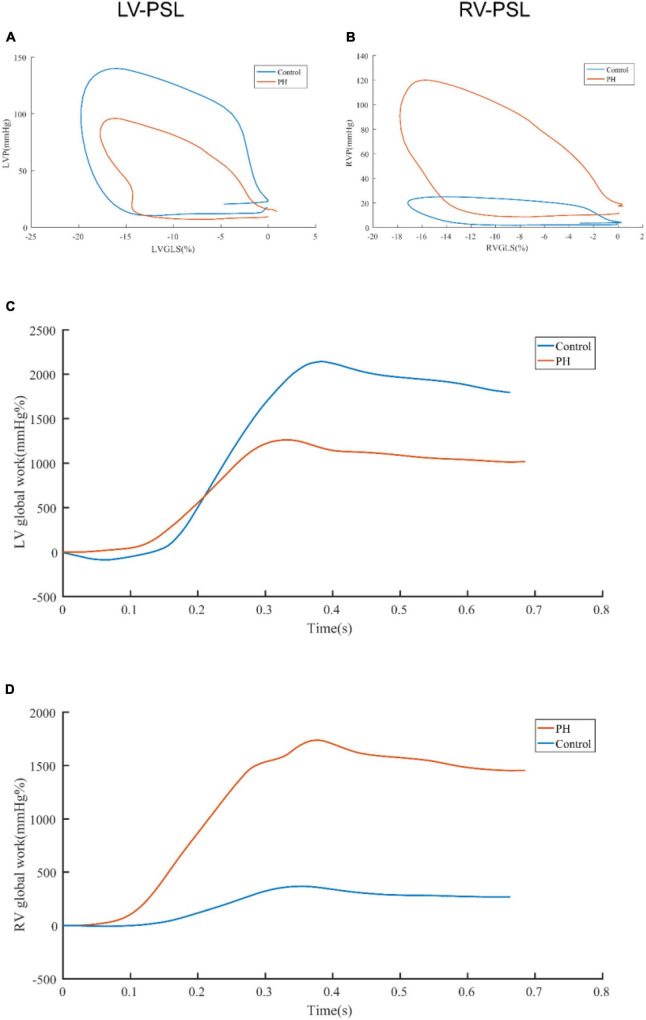
Representative pressure-strain loops and global work curves. The difference of LV-PSL **(A)** and RV-PSL **(B)** between PH patients and control was shown. The difference of LV global work curve **(C)** and RV global work curve **(D)** was shown. LV-PSL, left ventricular pressure-strain loop; RV-PSL, right ventricular pressure-strain loop.

The representative PSL and global work curves of participants were presented in Figure 3. As summarized in Table 3, PH patients had higher ePASP (74.0 ± 27.4 vs 25.2 ± 2.6 mmHg, *P* < 0.001), RAA (18.2 ± 6.6 vs 10.7 ± 2.0 cm^2^, *P* < 0.001), RV/LV (1.3 ± 0.4 vs 0.7 ± 0.1, *P* < 0.001), LVGLS (−15.3 ± 3.4 vs −19.3 ± 2.0%, *P* < 0.001), RVGLS (−13.5 ± 4.3 vs −21.8 ± 2.5%, *P* < 0.001) and lower TAPSE (16.5 ± 3.5 vs 24.0 ± 3.2 mm, *P* < 0.001), FAC (31.2 ± 12.2 vs 52.1 ± 6.7%, *P* < 0.001) than controls. However, there was no significant difference in LVEF (69.9 ± 5.7 vs 67.6 ± 3.9%, *P* = 0.075). Compared to the controls, LVGWW [296.5 (172.0, 461.5) vs 110.0 (83.0, 130.0), *P* < 0.001], RVGWI [571.5 (373.5, 796.0) vs 352.0 (314.0 412.0), *P* < 0.001], RVGCW [793.5 (624.5, 1,173.0) vs 474.0 (435.0, 526.0), *P* < 0.001] and RVGWW [151.5 (60.5, 223.0) vs 38.0 (23.0, 57.0), *P* < 0.001] were significantly increased in PH patients. LVGWE [84.0 (80.0, 90.0) vs 94.0 (93.0, 95.0), *P* < 0.001], LVGWI [1,330.0 (1,061.3, 1,467.5) vs 1,560.0 (1,364.0, 1,884.0), *P* < 0.001], LVGCW [1,810.0 (1,494.8, 2,024.5) vs 1,989.0 (1,795.0, 2,323.0), *P* = 0.004], and RVGWE [85.0 (76.3, 92.5) vs 92.0 (90.0, 95.0), *P* = 0.001] were significantly lower in PH patients than controls.

**TABLE 3:** Comparison of myocardial work parameters between pulmonary hypertension patients and controls.

Characteristic	Controls (*n* = 27)	PH (*n* = 52)	*P*-value
Gender (% female)	23 (85.2)	44 (84.6)	0.947
Age (years)	43.1 ± 13.7	44.1 ± 13.2	0.748
SBP (mmHg)	118.4 ± 13.8	120.4 ± 18.1	0.627
DBP (mmHg)	75.7 ± 10.3	75.7 ± 13.4	0.997
**Conventional echocardiography measurements**			
ePASP (mmHg)	25.2 ± 2.6	74.0 ± 27.4	< 0.001
TAPSE (mm)	24.0 ± 3.2	16.5 ± 3.5	< 0.001
FAC (%)	52.1 ± 6.7	31.2 ± 12.2	< 0.001
RAA (cm^2^)	10.7 ± 2.0	18.2 ± 6.6	< 0.001
RV/LV	0.7 ± 0.1	1.3 ± 0.4	< 0.001
LVEF (%)	67.6 ± 3.9	69.9 ± 5.7	0.075
LVGLS (%)	−19.3 ± 2.0	−15.3 ± 3.4	< 0.001
RVGLS (%)	−21.8 ± 2.5	−13.5 ± 4.3	< 0.001
**Myocardial work parameters**			
LVGWE (%)	94.0 (93.0, 95.0)	84.0 (80.0, 90.0)	< 0.001
LVGWI (mmHg%)	1,560 (1,364.0, 1,884.0)	1,330.0 (1,061.3, 1,467.5)	< 0.001
LVGCW (mmHg%)	1,989.0 (1,795.0, 2,323.0)	1,810.0 (1,494.8, 2,024.5)	0.004
LVGWW (mmHg%)	110.0 (83.0, 130.0)	296.5 (172.0, 461.5)	< 0.001
RVGWE (%)	92.0 (90.0, 95.0)	85.0 (76.3, 92.5)	0.001
RVGWI (mmHg%)	352.0 (314.0, 412.0)	571.5 (373.5, 796.0)	< 0.001
RVGCW (mmHg%)	474.0 (435.0, 526.0)	793.5 (624.5, 1,173.0)	< 0.001
RVGWW (mmHg%)	38.0 (23.0, 57.0)	151.5 (60.5, 223.0)	< 0.001

Values are mean ± SD, *n* (%), or median (first and third quartiles).

ePASP, estimation of pulmonary arterial systolic pressure; FAC, fractional area change; LVEF, left ventricular ejection fraction; RV/LV, right ventricle/left ventricle basal diameter ratio; TAPSE, tricuspid annular plane systolic excursion; other abbreviations as in Tables 1, 2.

### Relationship of myocardial work parameters and other clinical data in pulmonary hypertension patients

**FIGURE 4:**
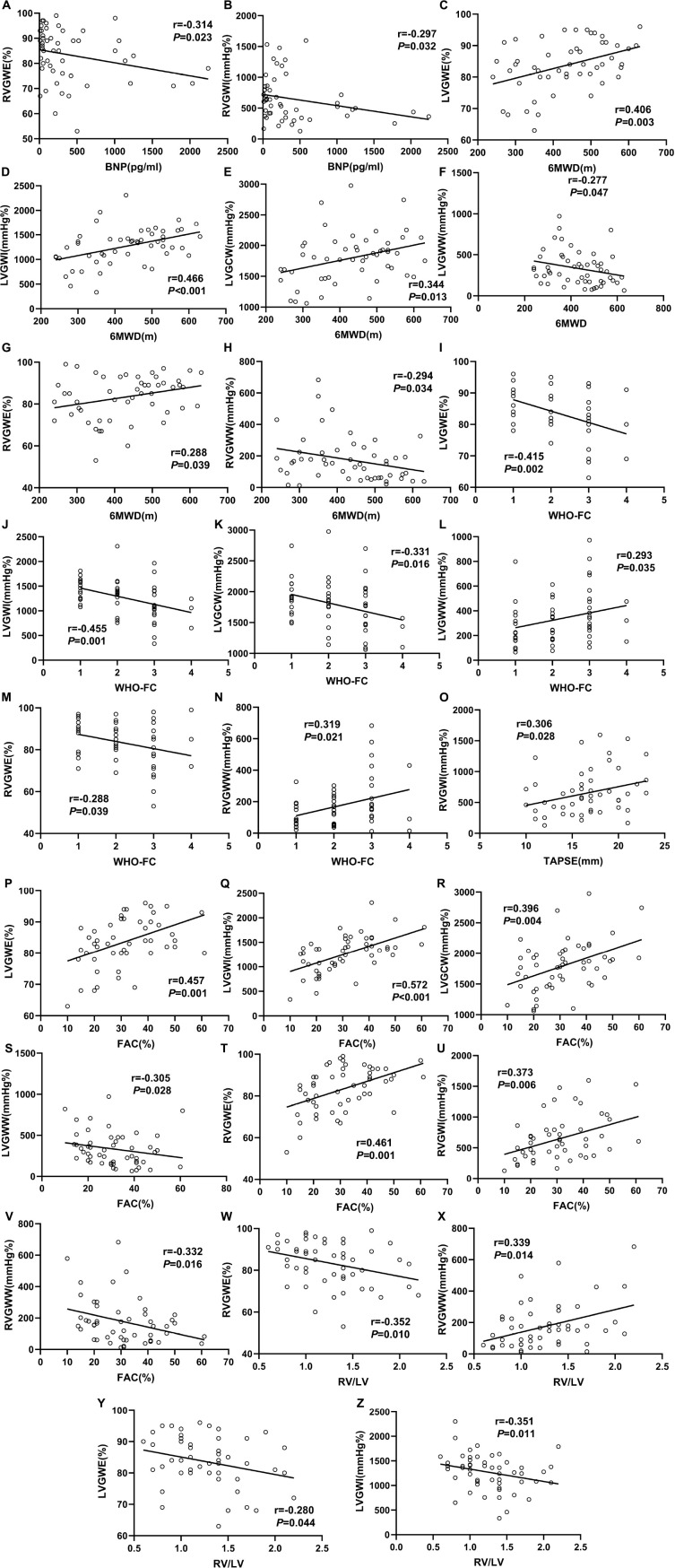
Correlation between clinical measurements and myocardial work parameters in the PH patients. The Spearmen correlation coefficients were tested to analyze the relationship between BNP **(A,B)**, 6MWD **(C–H)**, WHO-FC **(I–N)**, TAPSE **(O)**, FAC **(P–V)**, and RV/LV **(W–Z)** with myocardial work parameters. All abbreviations as in Tables 1, 2.

We used linear correlation analysis to assess relationship between myocardial work parameters and other clinical data in PH patients, which were described in Figure 4. LVGWE presented a positive correlation with 6MWD (*r* = 0.406, *P* = 0.003), and FAC (*r* = 0.457, *P* = 0.001), but a negative correlation with WHO-FC (*r* = −0.415, *P* = 0.002), and RV/LV (*r* = −0.280, *P* = 0.044). LVGWI significantly correlated with 6MWD (*r* = 0.466, *P* < 0.001), WHO-FC (*r* = −0.455, *P* = 0.001), FAC (*r* = 0.572, *P* < 0.001), RV/LV (−0.351, *P* = 0.011). The correlations between LVGCW and 6MWD (*r* = 0.344, *P* = 0.013), WHO-FC (*r* = −0.331, *P* = 0.016), and FAC (*r* = 0.396, *P* = 0.004) were also significant. LVGWW was only correlated with 6MWD (−0.277, *P* = 0.047), WHO-FC (*r* = 0.293, *P* = 0.035) and FAC (*r* = −0.305, *P* = 0.028). As for RV function, RVGWE showed a negative correlation with BNP (*r* = −0.314, *P* = 0.023), WHO-FC (*r* = −0.288, *P* = 0.039), and RV/LV (*r* = −0.352, *P* = 0.010) and a positive correlation with 6MWD (*r* = 0.288, *P* = 0.039), and FAC (*r* = 0.461, *P* = 0.001). In addition, RVGWI correlated well with BNP (*r* = −0.297, *P* = 0.032), TAPSE (*r* = 0.306, *P* = 0.028), and FAC (*r* = 0.373, *P* = 0.006). The correlations of RVGWW with 6WMD (*r* = −0.294, *P* = 0.034), WHO-FC (*r* = 0.319, *P* = 0.021), FAC (*r* = −0.332, *P* = 0.016) and RV/LV (*r* = 0.339, *P* = 0.014) were significant as well.

### Association of myocardial work parameters and clinical outcomes in pulmonary hypertension patients

**FIGURE 5:**
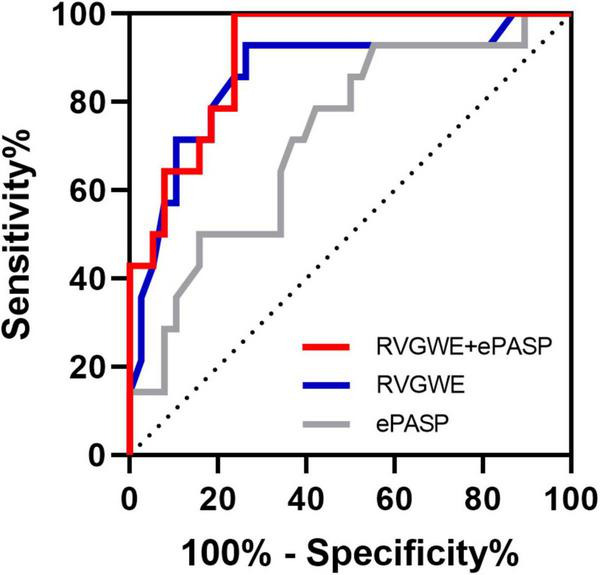
ROC curves for the prediction of adverse events. Major adverse events were defined as hospitalization and need of new specific drug therapy or enhancement on the original therapy basis. All abbreviations as in Tables 2, 3.

PH patients were followed for a median of 515.0 days (386.5, 535.8) at termination. During the follow-up period, adverse events occurred in 14 patients (26.9%): 12 patients (23.1%) had hospitalization and 2 patients (3.8%) needed enhancement on the basis of original therapy. Best binary logistic model for evaluating adverse events was shown in Table 4 (Hosmer-Lemeshow χ^2^ = 4.84, *P* = 0.775). The formula was as follows: logit (*P*) = 12.586 − 0.22*RVGWE + 0.051*ePASP. In the model, RVGWE had an OR of 0.803 (95%CI 0.698–0.922, *P* = 0.002) and ePASP had an OR of 1.052 (95%CI 1.010–1.096, *P* = 0.015). Furthermore, based on the model, ROC curves showed the combination of RVGWE and ePASP had the biggest area under curve (AUC) with 0.910 (*P* < 0.0001), which were described in Table 5 and Figure 5.

**TABLE 4:** Best binary logistic model for evaluating adverse events.

Variable	OR	95%CI	*P*-value
RVGWE (%)	0.803	0.698–0.922	0.002
ePASP (mmHg)	1.052	1.010–1.096	0.015

OR, odds ratio; other abbreviations as in Tables 2, 3.

**TABLE 5:** Receiver operating characteristic analysis for the prediction of adverse events.

Parameter	AUC	*P*-value	Sensitivity (%)	Specificity (%)
RVGWE + ePASP	0.910	< 0.0001	100.0	76.3
RVGWE (%)	0.861	< 0.0001	92.9	73.7
ePASP (mmHg)	0.719	0.016	92.9	44.7

AUC, area under curve; other abbreviations as in Tables 2, 3.

## Discussion

PSL was firstly proposed by Urheim et al. (16) to quantify regional myocardial function by combining LV pressure from micromanometer with myocardial longitudinal strains from strain Doppler echocardiography or sonomicrometry. On the basis of this, Russell et al. (10) established an improved non-invasive pressure curve and applied this to measure PSL area which was approximately equivalent to GWI. Furthermore, GCW, GWW and GWE derived from myocardial work assessment were put forward and measured to understand cardiac function more easily (14). Recent studies had shown that LVGWE, LVGCW, and LVGWW exhibited favorable applicability to predict response to cardiac resynchronization therapy (CRT) and long-term cardiac outcome in CRT candidates (17–21). Besides, it has proven that myocardial work parameters were superior to GLS to detect significant coronary artery disease in patients with no regional wall motion abnormalities and normal LVEF by Edwards et al. (22). Butcher et al. (23) also applied RV-PSL to assess RV function in a cohort of patients with heart failure with reduced left ventricular ejection fraction and found that RVGCW could reflect RV systolic function well and it correlated closely with invasively measured stroke volume and stroke volume index. Recently, Butcher et al. reported that decreased values of RVGCW and RVGWI were associated with all-cause mortality in patients with PH (24). This is the first study that quantifies myocardial work through PSL method to evaluate cardiac function and predict clinical prognosis in patients with PH.

Non-invasive PSL refers to estimated ventricular pressure and strain derived from echocardiography. According to Russell et al. (10), LV peak ventricular pressure can be substituted by systolic cuff pressure. Analogously, RV peak ventricular pressure should be equal to PASP from RHC. Echocardiography is widely used to measure non-invasive ePASP, and there is a good correlation between the estimation and actual value (25). A meta-analysis including 29 studies showed that the correlation coefficient between ePASP and PASP was 0.70 (26). However, the accuracy of ePASP is still questioned due to the effect of tricuspid regurgitation. In order to improve the agreement between PASP estimated from echocardiography and measured by RHC, PASPcal is generated by adjusting for TAPSE, FAC, RAP, RAA, RV/LV, LVGLS and RVGLS. It will be more accurate to apply PASPcal instead of ePASP to non-invasive PSL method to calculate RV myocardial work.

In our study, RVGWI, RVGCW and RVGWW substantially increase in PH patients compared to healthy controls while RVGWE decrease to a certain extent. RV is not composed of a single layer of myocardium, but mainly composed of superficial myocardium from the basal part of interventricular septum (IVS) and deep longitudinal myocardium. Under normal conditions, the contraction patterns of RV are mainly as follows: contraction of LV and IVS pull the free wall of RV to move inward, resulting in passive contraction of RV myocardium; the deep longitudinal myocardium of RV free wall contract, causing the tricuspid annular plane to approach to the apex (27); IVS rotates and contracts, participating in the shortening of RV long axis (28). With compensatory hypertrophy of the longitudinal myocardium of inner layer of RV in patients with PH, myocardial work increases to maintain RV ejection volume.

LV and RV are closely related in structure and function, and there is an interaction between two ventricles (29). In Hardegree et al. study (30), despite normal LV size and normal conventional measures of LV systolic function, including end-diastolic dimension, LVEF, and CI, patients with PH had reduced LV free wall systolic strain. The phenomenon may be explained by the reason that chronic RV pressure overload enlarges RV volume and forces IVS to deviate to LV, which further causes LV geometric deformation and dysfunction (31). This is supported by the results of the present study, in which PH patients have poorer LVGWE, LVGWI and LVGCW than healthy controls, whereas LVGWW abnormally increases in PH patients. Meanwhile, we also find that LVEF of patients was not significantly different from that of the control group, which may be due to compensatory enhancement of RV contractility to support LV systolic function (32).

In this study, as an emerging quantitative marker of cardiac function, myocardial work parameter has potential value to definite present cardiac function in PH patients. In clinical practice, WHO-FC remains a determinant part for assessing cardiac function, as it provides cardiologists with valuable information for determining disease severity, improvement, deterioration or stability. A follow-up cohort study containing 982 PH patients with WHO-FC III at baseline in the REVEAL Registry have shown that patients who improve from WHO-FC III to I/II have better prognosis than those who remain III or worsen to IV by Kaplan-Meier estimates of 3-year survival (33). WHO-FC presents substantial utility of evaluation of patients with PH. However, there is certain subjectivity and interobserver variation in WHO-FC assessment which is dependent on the experience of cardiologists (34). As supplements, myocardial stress markers such as BNP (35), exercise capacity tests such as 6MWD (36) and other echocardiography characteristics such as TAPSE (37), FAC (38), RAA (39), and RV/LV (40) are referred to evaluate cardiac function in PH patients more accurately. The NORRE study had proven LV myocardial work parameters correlate well with traditional parameters of systolic function in healthy subjects (41, 42). In the present study, myocardial work parameters correlate significantly with other clinical assessments of cardiac function. Myocardial work parameters will provide quantifiable information for identification of cardiac function status as another supplement to aid in clinical decision making.

Management of cardiac function is critical to the prognosis of patients with PH. Guidelines put emphasis on the importance of RV function in PH patients (1), as RV function has proven been a major determinant of prognosis among PH patients irrespective of etiology (43). RV is sensitive to pressure overload. And analysis of RV function independent of the effect of pulmonary artery pressure, does not provide accurate clinical evidence. RV-pulmonary artery coupling explains RV function in the perspective of the pulmonary circulation as a whole (44). There is no conclusive evidence for non-invasive assessment of RV-pulmonary artery coupling (45). In our current study, combination of RVGWE and ePASP is a potential novel model for assessment of RV-pulmonary artery coupling. RVGWE is a form of RV intrinsic contractility derived from PSL which is less dependent on the load. In addition, ePASP can reflect the elasticity of the pulmonary artery indirectly. In the prognostic study, the value of combination of RVGWE and ePASP is superior to individual parameter with the biggest AUC of 0.910. It is worthwhile to highlight that combination of RVGWE and ePASP has potential prognostic value to assist physicians in deliberating on the therapeutic schedule for each patient during follow-up.

There are some limitations in the present study. First, this is a single-center and small population cohort study, which may produce a selection bias. Because of a relatively short follow-up period, the prognostic use of myocardial work by PSL method needs to be further demonstrated. Second, in the absence of myocardial work analysis before specific drug therapy, we have no chance to explore the short- and long-term effect of specific drug therapy on myocardial work parameters.

## Conclusion

Non-invasive PSL is feasible for quantifying myocardial work in patients with PH. Myocardial work parameters derived from PSL method are the emerging markers of cardiac function and the combination RVGWE and ePASP is a useful predictor of the clinical outcome in patients with PH.

## Data availability statement

The original contributions presented in this study are included in the article/supplementary material, further inquiries can be directed to the corresponding author.

## Ethics statement

The studies involving human participants were reviewed and approved by Clinical Ethics Committee of Shanghai Renji Hospital. The patients/participants provided their written informed consent to participate in this study.

## Author contributions

JW: original manuscript drafting and writing—review and editing. CN: statistical analysis and figures graphing. MY and XZ: data collection and literature research. BR and LS: clinical studies. XS and JS: conceptualization, supervision, project administration, and funding acquisition. All authors have studied concepts/study design or data acquisition or data analysis/interpretation, manuscript drafting or manuscript revision for important intellectual content, approval of final version of submitted manuscript, and agreed to ensure any questions related to the work are appropriately resolved.
